# Acute Panmyelosis with Myelofibrosis - A Rare Subtype of Acute Myeloid Leukemia

**DOI:** 10.4084/MJHID.2013.042

**Published:** 2013-06-04

**Authors:** Tathagata Chatterjee, Srishti Gupta, Ajay Sharma, Sanjeevan Sharma, Devika Gupta

**Affiliations:** 1 Department of Pathology and Molecular Medicine, Army Hospital (Research and Referral), Delhi Cantt - 110010 India; 2 Department of Immunohematology and Transfusion Medicine, AFMC, Pune - 411040 India

## Abstract

One case of acute panmyelosis with myelofibrosis (APMF) is here reported. A 45 year old male presented with abrupt onset of rapidly progressing low backache, weakness and pancytopenia. On examination there was no organomegaly. Peripheral blood examination revealed normocytic normochromic red blood cells with 10% circulating blasts. Flowcytometric examination of peripheral blood revealed blasts which were positive for CD34, HLA− DR and myeloid associated antigens (i.e. CD13 and CD33). Blasts were negative for anti MPO. Bone marrow aspirate resulted in a dry tap. Bone marrow biopsy revealed panmyeloid proliferation with scattered blasts which were CD34 positive on imunohistochemistry and negative for anti MPO. Reticulin stain showed grade III myelofibrosis (WHO). Differential diagnosis considered included AML-M7, MDS-RAEB II and AML with myelodysplasia. He was started on chemotherapy [idarubicin and cytarabine; 3+7 induction regimen followed by three cycles of HIDAC (High dose cytosine arabinoside)] after which patient was in complete morphological remission with markedly reduced bone marrow fibrosis. He is now being worked up for allogeneic stem cell transplantation. Patient is asymptomatic at eight months of diagnosis. In conclusion these patients should be managed aggressively with AML therapy and this case report reaffirms the fact that APMF is subtype of AML.

## Introduction

Acute panmyelosis with myelofibrosis (APMF) is a rare form of acute myeloid leukemia and is characterized by acute panmyeloid proliferation with increased blasts and accompanying fibrosis of the bone marrow that does not meet the criteria for AML with myelodysplasia related changes.^1^

APMF is classified under acute myeloid leukemia not otherwise specified by WHO 2008.^1^ This entity is distinct and needs to be distinguished from acute megakaryoblastic leukemia (AML-M7), myelodysplastic syndrome - refractory anemia with excess blast II ( MDS-RAEB-II) with fibrosis, primary myelofibrosis (PMF) and AML with myelodysplasia related changes.^2^ The clinical course of this entity is rapidly progressive and fatal, therefore, it is essential to be aware of this entity and distinguish it from its mimickers.^1^,^2^ Though it may be extremely difficult to differentiate APMF from its mimickers in some cases, detailed clinical history and hematological work up can be helpful in such cases. Many consider, as evidenced by many published articles, that APMF is a variant of MDS.^3^,^4^,^5^,^6^ Since the outcome of these patients is poor therefore it is important to aggressively manage these patients with timely diagnosis as it can reduce morbidity and prolong life. In this case report other differential diagnosis considered are highlighted.

## Case presentation

A 45 year old male presented with acute onset rapidly progressive low backache, fatigue and weakness for past one month. On examination he had no organomegaly, lymphadenopathy, petechiae or purpura. There was no significant past and family history. There was no history of any cytotoxic drug intake. Peripheral blood examination revealed pancytopenia. Total leukocyte count was 3000/mm^3^ with a platelet count of 40000/mm^3^ and hemoglobin level of 7 gm/dl. Red blood cells were normocytic normochromic with minimal anisopoikilocytosis. Eight nucleated red blood cells were seen per hundred white blood cells. Occasional fully chromic tear drop cells were also noted. Differential leukocyte count comprised of blast 10%, polymorphonuclear leukocyte 65%, lymphocyte 23%, eosinophil 02%, basophil 0%. Blasts morphologically were myeloblast with an occasional auer rod. However cytochemical MPO stain on peripheral blood smear was negative.

**Figure 1:**
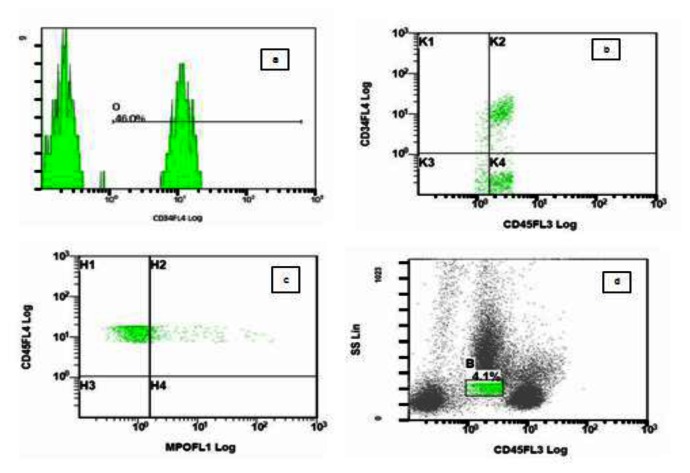
Figure 1 shows flowcytometric analysis on peripheral blood of CD34 antigen on blast population (a) shows histogram of CD34+blasts (B) shows scatter diagram of CD34 vs CD 45. Blasts are CD34 + and CD45 + (c) shows scatter diagram of CD 45 vs cMPO. Blasts are negative for cytoplasmic myeloperoxidase (d) Figure shows 4.1% blasts gated using CD45 vs SSC gating strategy

Flow cytometric analysis was done on peripheral blood using Beckman Coulter FC 500 flowcytometer (Beckman Coulter, Miami, FL, USA). Gating strategy was SSC vs CD45. An antigen was considered positive if greater than 20% of blasts gated showed positivity for a specific monoclonal antibody. Antibodies used were CD19, CD10, CD34, anti-MPO, HLA DR, CD11c, CD14, CD4, CD8, CD3, cCD79a, cCD3, CD117, CD15, CD13, CD33 and CD7. Antibodies against CD41, CD61, glycophorin A could not be used as they were not available at that time. 4.1% blasts were gated which were CD45 positive (moderate) and had low side scatter. 46% of gated blasts were positive for CD34 (Figure 1). Flow cytometry result is shown in table 1.

**Figure 2:**
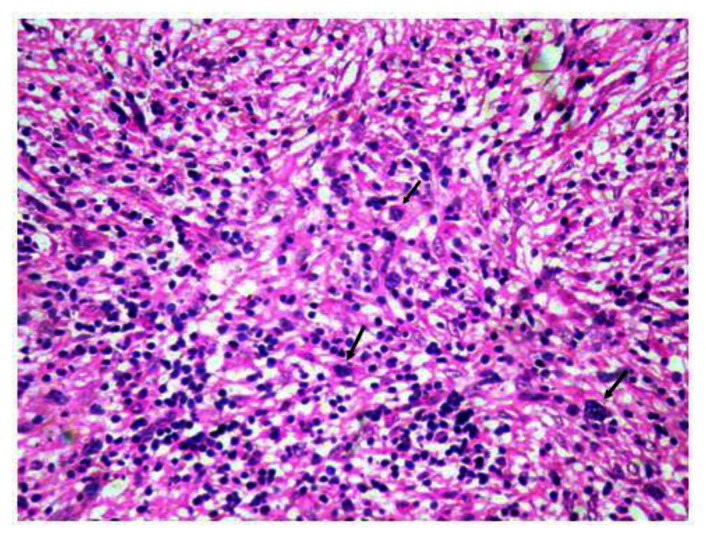
Figure 2 shows panmyeloid proliferation with dyspoietic unilobated megakaryocytes(arrow). (Hematoxylin and eosin × 40x).

**Figure 3:**
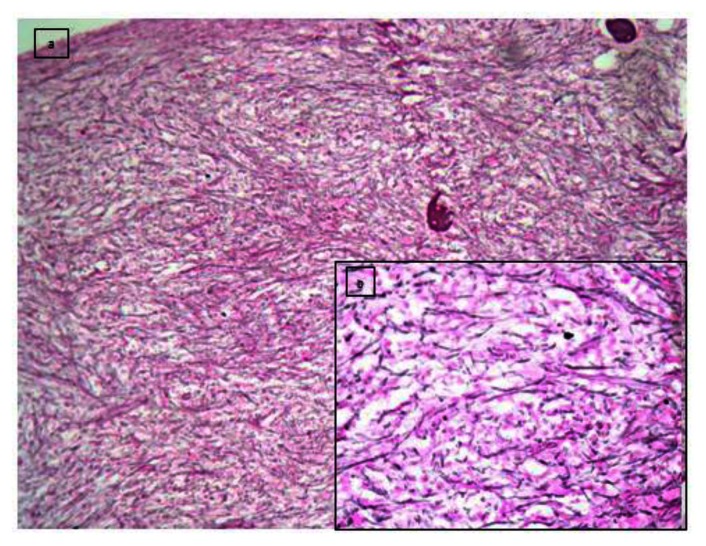
a & b (inset) shows increased WHO grade III fibrosis on reticulin stain(Gomori silver impregnation technique(a-10x)(b-40x).

Bone marrow aspirate was unsuccessful but bone marrow biopsy (Figure 2) revealed hypercellular bone marrow with panmyeloid proliferation. Dyspoietic megakaryocytes, immature myeloid precursors and erythroid precursors were seen. Numerous small megakaryocytes were noted with hypolobated and nonlobated nuclei and dispersed chromatin. Blasts were seen dispersed as well as in clusters. However no atypical localization of immature precursors was seen. Reticulin stain revealed a characteristic WHO grade III myelofibrosis^7^ (Figure 3).

**Figure 4:**
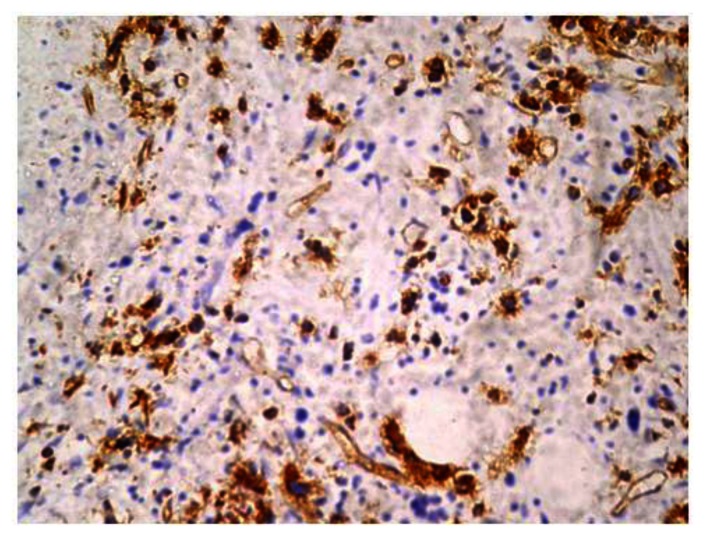
Figure 4 shows CD 34 positive blasts with a working internal control (arrow-endothelium) (10x) (DAKO automated immunostainer-streptavidin biotin - peroxidase based technique).

Immunohistochemistry was done on paraffin embedded sections using automated immunostainer (DAKO, CA, USA) based on standard streptavidin biotin-peroxidase complex technique. Antibodies used were antiCD61, anti glycophorin A, antiCD34 and anti MPO. Blasts were positive for CD34 (Figure 4) and negative for CD61, anti MPO and glycophorin A.

JAK2 mutation analysis was carried out and revealed a wild type mutant. Conventional cytogenetic analysis was carried out to rule out any abnormal cytogenetics. However no abnormality was detected.

The patient was treated as a case of acute myeloid leukemia with a 3+7 induction regimen of idarubicin (12 mg/m^2^) and cytarabine (100 mg/m^2^). This was followed by three consolidation cycles of HIDAC (High dose intermittent ARA-C; each cycle comprising of 4.5 g BD for three days) which led to marked improvement in symptoms. Bone marrow biopsy done for remission status after 45 days of starting HIDAC regimen revealed a dramatic reduction in fibrosis to grade I. Blasts were reduced to less than 5%. Patient is being planned for autologous hematopoietic stem cell transplant due to non-availability of HLA-matched donor.

## Result

The outcome of such patients is usually poor with a rapidly fatal course. Above patient has responded to chemotherapy as evidenced by symptomatic improvement, decrease in blast percentage to less than 5% and reversal of myelofibrosis from grade III to grade I. Patient is asymptomatic at eight months of diagnosis and has not had any episode of relapse.

## Discussion

Differential diagnosis entertained in this case included AML-M7, MDS-RAEB-2 with fibrosis, PMF and AML with myelodysplasia related changes. In AML-M7 blasts are more than 20% of all nucleated cells in peripheral blood and/or bone marrow and atleast 50% are megakaryoblasts (CD41/CD61 positive).^1^ This was not so in our case. Additionally blasts in APMF are always positive for CD34 and in AML M7 blasts are positive for CD34 only in 60% of the cases.^2^

It was difficult to differentiate APMF from MDS-RAEB II with fibrosis and this difficulty has been evidenced by many published manuscripts.^2^,^5^,^8^ MDS-RAEB II with fibrosis was negated based on abrupt clinical presentation and immunohistochemistry result as blasts in our case were anti MPO negative. Also numerous small megakaryocytes with nonlobated and hypolobated nuclei with dispersed chromatin pointed towards a diagnosis of APMF. Lastly, the striking degree of myelofibrosis was also in favour of APMF.^2^

PMF was ruled out as there was no intrasinusoidal hematopoiesis. Also, characteristic tight cellular clusters of large megakaryocytes with clumped, hyperchromatic nuclei (cloud or balloon shape) were absent.^9^ AML with myelodysplasia related changes was ruled out as blasts were less than 20%.^10^

In view of abrupt onset of rapidly progressive symptoms in our case with panmyelosis a diagnosis of APMF was given.

APMF is a rare and aggressive form of AML and arises denovo.^1^ Many believe it responds poorly to chemotherapy and mean survival is 9 months.^1^ In our case aggressive management with AML chemotherapy gradually improved pancytopenia and reduced bone marrow fibrosis. This case highlights the importance of identifying and differentiating APMF from other entities like AML-M7 and RAEB-2 so that aggressive treatment can be instituted. This case also confirms that APMF is a type of AML unlike many who believed it to be a type of MDS.^3^,^4^,^5^,^6^

To conclude, in a patient with an abrupt onset of fever, bone pain and pancytopenia a diagnosis of APMF should be kept in mind. Such patients are usually adults with no organomegaly. The bone marrow (BM) tap is usually dry due to extensive fibrosis and BM biopsy features are characteristic. Panmyelosis with poorly differentiated blasts and scattered hypolobated and nonlobated megakaryocytes are noted. Immunophenotypic analysis usually shows blasts to be CD34, CD13, CD33 and CD 117 positive and negative for megakaryocytic lineage and myeloperoxidase. Cytogenetic analysis helps to differentiate APMF from its mimickers specially MDS RAEB2 and acute megakaryoblastic leukemia.^1^

## Figures and Tables

**Table 1:** Flowcytometric result of peripheral blood sample, Gating strategy-SSC Vs CD45, Beckman Coulter FC 500, Events-50000.

Antibody	Result (Percentage positivity)	Intensity
CD34	46%	Dim to Moderate
CD13	55%	Dim to Moderate
CD33	66.3%	Dim to Moderate
CD45	78.9%	Moderate
HLA-DR	45.9%	Dim to moderate
CD117	16.5%	Dim
CD4	46%	Dim to moderate
CD19	2.9%	Negative
CD10	2.5%	Negative
CD15	21.7%	Strong
CD7	1.6%	Negative
CD3	0.2%	Negative
CD8	0.4%	Negative
Anti cMPO	5.5%	Negative
CD14	1.4%	Negative
CD11c	9.7%	Negative
cCD79a	8.4%	Negative
cCD3	0.2%	Negative
